# A comprehensive transcriptomic analysis of differentiating embryonic stem cells in response to the overexpression of Mesogenin 1

**DOI:** 10.18632/aging.101049

**Published:** 2016-10-06

**Authors:** Dahai Liu, Qiang Zhang, Hong Zhang, Ling Tang, Wei Li, Dongming Zhang, Guoying Wu, Shoudong Ye, Qian Ban, Kan He

**Affiliations:** ^1^ Center for Stem Cell and Translational Medicine, School of Life Sciences, Anhui University, Hefei City, Anhui 230601, P. R. China; ^2^ Department of Biostatistics, School of Life Sciences, Anhui University, Hefei City, Anhui 230601, P. R. China

**Keywords:** somitogenesis, microarray, pathway, embryonic stem cells, Msgn1

## Abstract

The mutation of somitogenesis protein Mesogenin 1 (Msgn1) has been widely used to study the direct link between somitogenesis and the development of an embryo. Several studies have used gene expression profiling of somitogenesis to identify the key genes in the process, but few have focused on the pathways involved and the coexpression patterns of associated pathways. Here we employed time-course microarray datasets of differentiating embryonic stem cells by overexpressing the transcription factor Msgn1 from the public database library of Gene Expression Omnibus (GEO). Then we applied gene set enrichment analysis (GSEA) to the datasets and performed candidate transcription factors selection. As a result, several significantly regulated pathways and transcription factors (TFs), as well as some of the specific signaling pathways, were identified during somitogenesis under Msgn1 overexpression, most of which had not been reported previously. Finally, significant core genes such as Hes1 and Notch1 as well as some of the TFs such as PPARs and FOXs were identified to construct coexpression networks of related pathways, the expression patterns of which had been validated by our following quantitative real-time PCR (qRT-PCR). The results of our study may help us better understand the molecular mechanisms of somitogenesis in mice at the genome-wide level.

## INTRODUCTION

In vertebrates, somites give rise to the axial skeleton, skeletal muscle, and dorsal dermis, which arose from the presomitic mesoderm(PSM), a mesoderm-derived tissue lying on both sides of the neural tube [1, 2]. The PSM is dynamic: somites are generated at the anterior end, each posterior PSM cell moves from its original position toward anterior PSM and is finally integrated into the somites [3, 4]. The clock and wavefront model has been established to make the clock mechanism that somitogenesis relies on well known. Molecular evidence for the existence of this clock has been obtained on the basis of the periodic expression of several genes related to Notch signaling in the PSM.

The segmentation clock is considered to play an important role in the periodic activation of Notch signaling in the PSM [5].

In vertebrate embryos, somite segmentation is controlled by the molecular clock mentioned above, in the form of a transcriptional oscillator that operates in the PSM. Mesogenin 1 *(Msgn1)*, a basic-Helix–Loop–Helix (bHLH) transcription factor that lay in the PSM [6, 7], is essential for differentiation, movement and maturation of PSM progenitor cells during somito-genesis [8]. *Msgn1* is expressed in the mesodermal compartment of the primitive streak and is necessary for the proper development of the mesoderm. *Msgn1* drives the changes in gene expression in the nascent PSM cells and regulates the movements from the posterior region to the PSM. Besides, *Msgn1* controls the size of somites and manipulates the size and existence of the progenitor cells. *Msgn1 acts as a* direct target gene of the Wnt/β-catenin signaling pathway, which can activate Notch signaling genes to control the segmentation clock. *Msgn1* can mediate crosstalk between the Wnt and Notch signaling pathways during mammalian somito-genesis [9]. Thus, *Msgn1* is a pivotal ingredient of a transcriptional cascade and plays a fundamental role in the segmentation clock. *Msgn1* in the differentiating embryonic stem cells can overexpress in the presence of Doxycycline(Dox) which is a member of the tetracycline antibiotics group. Thus, we used Dox to verdict the function of *Msgn1*.

*Msgn1* can activate the expression of numerous genes including cyclic genes in Notch signaling pathway via Wnt signaling program, which belongs to a deeply conserved cell signaling system present in most multicellular organisms [10]. Notch signaling cascade is composed of a conserved family of extracellular ligands and two cellular factors that may associate with the Notch intracellular domain. Binding of ligand proteins to the extracellular domain can induce proteolytic cleavage and release of the intracellular domain, which activates some target genes, such as *Lfng* and *Hes7* [11]. It has been known that Notch signaling is of capital importance in embryogenesis, central nervous system development and function, cardiovascular development and endocrine development. In this project, we focus on its central role to somitogenesis. In 1995, Conlon RA et al found that Notch1 would play important roles in the coordination of the somites segmentation in mice [12]. Further studies have identified the role of Notch signaling in the segmentation clock mentioned above.

Although the pivotal function of *Msgn1* in the segmentation clock has been revealed in previous studies, the multiple related pathways and associated transcription factors (TFs) were little known, especially in genome-wide. Here we employed time-course microarray datasets of differentiating embryonic stem cells by overexpressing the transcription factor Msgn1 from the public database library of Gene Expression Omnibus (GEO). Then we applied gene set enrichment analysis (GSEA) on the datasets and performed candidate transcription factors selection to explore the molecular mechanisms of somitogenesis in mice at the genome-wide level.

## RESULTS AND DISCUSSION

### Overview of the significantly regulated pathways and TFs during somitogenesis under Msgn1 overexpression

According to our GSEA of the data sets of 18 samples used in the microarray experiment by the comparison of Msgn1 overexpression to wild-type mice, there were 100 significantly associated pathways identified with p < 0.01 at 12h, including 83 upregulated and 17 down-regulated pathways. Moreover, there were 113 significant pathways including 29 upregulated and 84 downregulated ones at 24h, as well as 183 significant pathways including 50 upregulated and 113 down-regulated ones at 48h. The details of each pathway are given in Table S-1. In order to predict TFs potentially involved in embryonic stem cell differentiation in the case of *Msgn1* overexpression, we implemented the analysis of TFBSs and the prediction of TFs using the significant genes in each identified pathway. Based on the cutoff value of TF importance, we have identified the associated TFs with potential target genes that are co-regulated in each of the above-mentioned 376 pathways. The details are shown in Table S-2.

**Figure 1:**
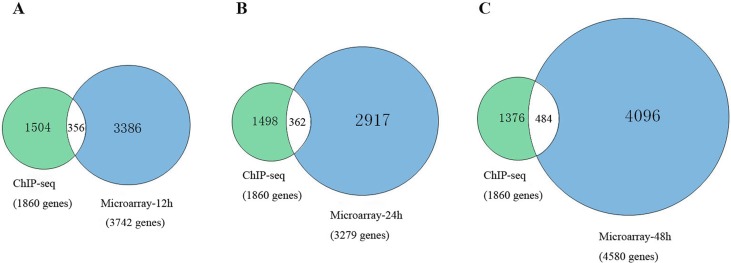
The comparison between related ChIP-seq results with our GSEA target genes These studies supposed that the major function of Notch signaling does not act upon a single cell, but coordinates cell clocks and keep them timed. This hypothesis illustrated the function of Notch signaling during segmentation and has been supported by experiments in mice and zebrafish [13, 14[. In vertebrates, Notch signaling is in the direct regulation of segmentation. Tight control of Notch signaling was crucial for the proper implementation of somitogenesis [15]. Notch signaling keeps the oscillations of neighbouring presomitic mesoderm cells synchronized in somite segmentation [16].

To further explain the regulatory mechanism of *Msgn1* functions in the process of embryonic stem cells development, we have further combined the related ChIP-seq results with our GSEA target genes in different developmental stages, which were shown in Figure 1. As a result, there were 356 common genes identified at 12h, 362 common genes at 24h and 484 common genes at 48h. The details of common genes are shown in Table S-3.

### Specific signaling pathways during somitogenesis under Msgn1 overexpression

**Figure 2:**
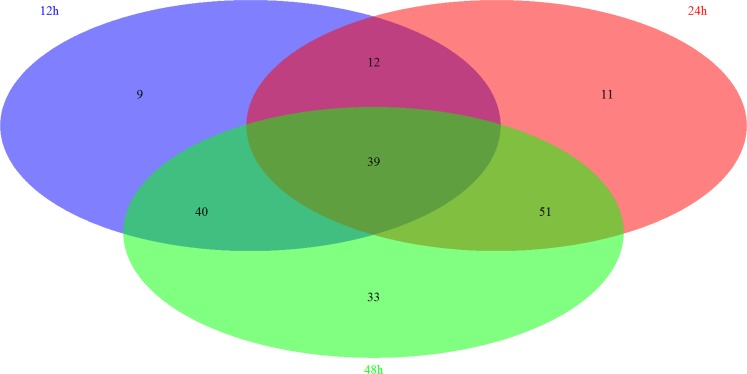
The summary of significantly regulated pathways based on GSEA under Msgn1 overexpression The Venn diagram showed the comparisons of each time point. There are 100 most significant pathways at 12 hour, 113 significant pathways at 24 hour and 163 significant pathways at 48 hour. By the comparison of 3 different time points, 39 significantly associated pathways were overlapped. By the comparison of 12h and 24h time points, 51 significant pathways were overlapped. Similarly, 79 and 90 overlapping significant pathways were identified respectively while comparing between 12h and 48h, between 24h and 48h.

Based on the GSEA approach, we have identified several significantly related pathways during somito-genesis in mice. By the comparison of each group, we have further identified 39 overlapping pathways significantly regulated in these three periods, including 8 signaling pathways (Figure 2). Most of these signaling pathways are related to immune system (3/8) and signal transduction (4/8) according to KEGG pathway maps in the KEGG database (www.genome.jp/kegg/). We next screened and reorganized the entire signaling pathways at three time points, the details of which were showed in Table S-4 and Table S-5. Fc epsilon RI signaling pathway is only significantly regulated in 12h, which belongs to the immune system. There are 2 specific signaling pathways significantly regulated in 24h, PPAR signaling pathway and Insulin signaling pathway, and both of them are concerned with the endocrine system. Similarly, there are 2 signaling pathways significantly regulated in 48h: Jak-STAT signaling pathway which is correlated with signal transduction and B cell receptor signaling pathway which is correlated with the immune system. Moreover, 2 signaling pathways play roles at both 12h and 24h, which are related to the nervous system and signal transduction respectively. In addition, 3 signaling pathways are regulated significantly at 12h and 48h. Among them, VEGF signaling pathway is related to the signal transduction, while Toll-like receptor signaling pathway and NOD-like receptor signaling pathway are belong to the immune system. In 24h and 48h overlapping signaling pathways, 3 signaling pathways functioned in the signal transduction while the other 2 signaling pathways act in the endocrine system.

According to Table 1, most signaling pathways are concerned with signal transduction (10/23) such as MAPK signaling pathway, Hedgehog signaling pathway and mTOR signaling pathway. Mitogen-activated protein kinases (MAPKs) signal is one of the important ways in the eukaryotic cell, which adjusts and controls the structure and function of the cell. MAPKs play a vital role in the production of matrix metalloproteinases and the regulation of cartilage cell proliferation, apoptosis and differentiation [17]. The Hedgehog pathway is a key signaling pathway controlling key steps of embryonic development and patterning through the early stages of development as well as in the development of most tissues and organs in mammals [18, 19]. MTOR is a core protein in early embryo development, which highlights the dynamic role of TOR signaling and presents additional functions beyond cell growth control during embryonic development [20]. In addition, some other signaling pathways like RIG-I-like receptor signaling pathway and Fc epsilon RI signaling pathway regulate in the immune system (7/23). The RIG-I-like receptor signaling pathway can help create the ability to recognize virus infection and to enhance a powerful antiviral response, which plays an important role in immune systems. RIG-I-like receptor signaling pathway can activate the production of antiviral cytokines and the establishment of an effective cellular antiviral state by triggering signal transduction pathways, which preserves neighboring cells against infection and initiates native and adaptive immune systems [21].

**Table 1:** The signaling pathways significantly down or up-regulated in 12h, 24h and 48h during somitogenesis

Signaling pathway	Functional class	12h	24h	48h
p53 signaling pathway	Cell growth and death	↑	↑	↓
GnRH signaling pathway	Endocrine system		↓	↓
Adipocytokine signaling pathway	Endocrine system		↓	↓
PPAR signaling pathway	Endocrine system		↑	
Insulin signaling pathway	Endocrine system		↓	
Chemokine signaling pathway	Immune system	↑	↓	↓
RIG-I-like receptor signaling pathway	Immune system	↑	↑	↓
T cell receptor signaling pathway	Immune system	↑	↓	↓
Toll-like receptor signaling pathway	Immune system	↑		↓
NOD-like receptor signaling pathway	Immune system	↑		↑
Fc epsilon RI signaling pathway	Immune system	↑		
B cell receptor signaling pathway	Immune system			↓
Neurotrophin signaling pathway	Nervous system	↑	↓	
VEGF signaling pathway	Signal transduction	↑		↓
Calcium signaling pathway	Signal transduction	↓	↓	↓
Notch signaling pathway	Signal transduction	↓	↓	↑
Hedgehog signaling pathway	Signal transduction	↓	↓	↓
ErbB signaling pathway	Signal transduction	↑	↓	
MAPK signaling pathway	Signal transduction	↑	↓	↓
mTOR signaling pathway	Signal transduction		↓	↓
Wnt signaling pathway	Signal transduction		↓	↓
TGF-beta signaling pathway	Signal transduction		↓	↓
Jak-STAT signaling pathway	Signal transduction			↓

Here, all of the signaling pathways at 12h, 24h and 48h during somitogenesis were shown. We compared the signaling pathways with KEGG pathway maps, and obtained the functional classification of these signaling pathways presented in second column. Finally, we performed the down or up-regulated information of each signaling pathways at each time points.

Notch signaling pathway is down-regulated in 12h and 24h, while it is upregulated in 48h. Wnt signaling pathway is down-regulated in 24h and 48h (shown in Table 1). It has been reported that chondrogenesis could be inhibited by Wnt/β-catenin signaling pathway and Wnt/β-catenin signaling pathway would be down-regulated during chondrogenesis under the induction of TGF-β [22]. We presume that Notch signaling pathway may play a vital role in the later stage of somitogenesis. While in the middle and later periods, Wnt signaling pathway may reduce its effects on somitogenesis.

### Network construction and dynamic regulation during somitogenesis

**Figure 3:**
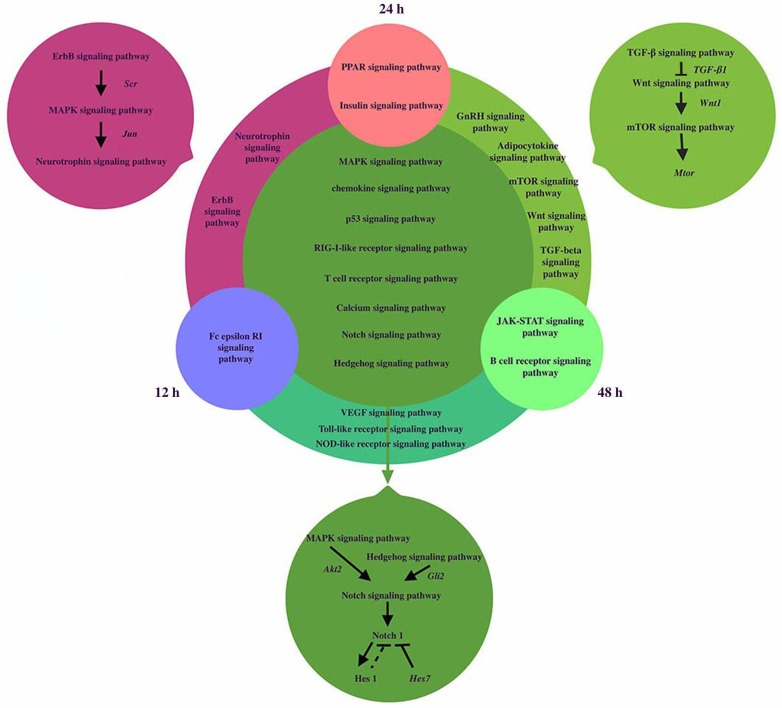
The coexpression networks of related pathways during somitogenesis We analyzed the signaling pathways network of dynamic regulation during somitogenesis at three different time points including12h, 24h and 48h. Fc epsilon RI signaling pathway was only significantly regulated in 12h, PPAR signaling pathway and Insulin signaling pathway were significantly regulated in 24h, Jak-STAT signaling pathway and B cell receptor signaling pathway were significantly regulated in 48h. 2 signaling pathways played roles at both 12h and 24h. Erb signaling pathway can activate Neurotrophin signaling pathway via MAPK signaling pathway. Jun, regulated by MAPK signaling pathway, induce neurotrophin activation. 5 signaling pathways regulated significantly at 24h and 48h, TGF-β signaling pathway activated Wnt signaling pathway via the expression of TGF-β1. Wnt1 is target gene of Wnt signaling pathway and Wnt1 can induced the activation of mTOR signaling pathway. 3 signaling pathways regulated significantly at 12h and 48h. 9 signaling pathways regulated at three different time points. MAPK and Hedgehog signaling pathways can activate Notch signaling pathway by activating Akt2 and Gli2 respectively. Notch1 and Hes1 are the downstream effectors of Notch signaling pathway and Hes1 is the target gene of Notch1. Hes7 can inhibit Notch target genes, so we hypothesize that Hes1 may have the similar effect. For simplicity, several target genes, gene products, and regulatory interactions are not shown.

The signaling pathways network was reconstructed to reveal the dynamic regulation during somitogenesis at three different time points including12h, 24h and 48h (shown in Figure 3). MAPK and Hedgehog signaling pathways can activate Notch signaling pathway by activating *Akt2* and *Gli2* respectively [23, 24[. Inhibition of the MAPK signaling pathway decreases the expression of *Notch1* and *Hes1*. *Notch1* and *Hes1* are downstream effectors of Notch signaling pathway and *Hes1* is the target gene of *Notch1* [25, 26]. *Hes7*, a segmentation clock, can inhibit Notch target genes, so we hypothesize that *Hes1* may have the similar effect. Besides, the relationship of genes and pathways specially involved in 24h and 48h was showed in Figure 2. TGF-β signaling pathway activated Wnt signaling pathway via the expression of *TGF-β1* [22, 27]. *Wnt1* is target gene of Wnt signaling pathway and *Wnt1* can induce the activation of mTOR signaling pathway [28].

Members of the Transforming Growth Factor-beta (TGFβ) superfamily of cytokines play crucial roles in pluripotency and differentiation of embryonic stem cells in vitro, which are essential for early mammalian embryonic development [29]. mTOR signaling pathway can influence muscle development in mice by majoring the accumulation of protein level in somites [30].

Erb signaling pathway can activate Neurotrophin signaling pathway via MAPK signaling pathway. The ErbB signaling pathway regulates proliferation, differentiation, cell motility, and survival. MAPK pathway is a downstream target of ErbB receptors. The highly conserved module of MAPK cascade participates in a variety of cellular functions, including cell proliferation, differentiation and migration. Neuro- trophins belong to the category of trophic factors, which are involved in the differentiation, growth and survival of cells [31]. Several intracellular signaling pathways including the MAPK cascade would be stimulated by the activation of multiple ligands such as the epidermal growth factor (EGF) on ErbB receptor, which is regulated by Src [32]. From Jun Yamauchi et al.'s study, *Jun*, regulated by MAPK signaling pathway, induces neurotrophin activation [33]. All the genes mentioned above can be found in Tables 4, and they confirm our analysis once again.

**Figure 4:**
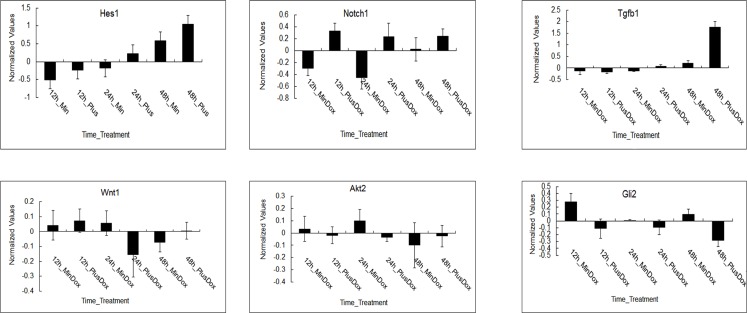
The expression patterns of regulated genes in major signaling pathways during somitogenesis under Msgn1 overexpression Microarray analysis of of gene expression in the absence and presence of Dox over a 48 hour timecourse. log2 ratios of the normalized expression levels of Hes1, Notch1, Tgfβ1, Wnt1, Akt2 and Dli2 are presented. The expression of Hes1 and Tgfβ1 was upregulated under Msgn1 overexpression over a 48 hour timecourse. Notch1 expression was variable, presumably reflecting the dynamic expression of a cyclic gene, but was generally elevated by Msgn1 overexpression. Tgfβ1 expression was upregulated in treated groups compared with untreated ones at 24h, while Wnt1 expression was just opposite. It may confirm that TGF-β signaling pathway could activate Wnt signaling pathway via the expression of Tgfβ1. Notch1 and Hes1 expressions were both upregulated in the treated groups compared with untreated ones from 12h to 48h. The expression of Akt2 and Dli2 were both downregulated compared with control group while Notch1 expression was upregulated in treated groups, presumably reflecting other signaling pathways, Wnt signaling pathway for example, activate Notch signaling pathway. Here error bars indicate standard deviation of 3 biological replicates.

Log2 ratios of the normalized expression levels of *Hes1*, *Notch1*, *Tgfβ1*, *Wnt1*, *Akt2* and *Dli2* are presented (shown in Figure 4). Error bars demonstrate standard deviation of 3 biological replicates. The expression of *Hes1* and *Tgfβ1* was upregulated under Msgn1 over-expression over a 48-hour time course. The expression of *Notch1* was variable, presumably reflecting the dynamic expression of a cyclic gene, but was generally elevated by Msgn1 overexpression. *Tgfβ1* expression was upregulated in treated groups compared with untreated ones at 24h, while *Wnt1* expression was just opposite, which confirms Tgfβ signaling pathway can activate Wnt signaling pathway via the expression of *TGF-β1*. The expressions of *Notch1* and *Hes1* were upregulated in treated groups compared with untreated ones from 12h to 48h, which was consistent with results shown in Figure 4. The expressions of *Akt2* and *Gli2* were downregulated compared with the control group while the *Notch1* expression was upregulated in treated groups, presumably reflecting other signaling pathways. The Wnt signaling pathway, for example, activates the Notch signaling pathway.

**Figure 5:**
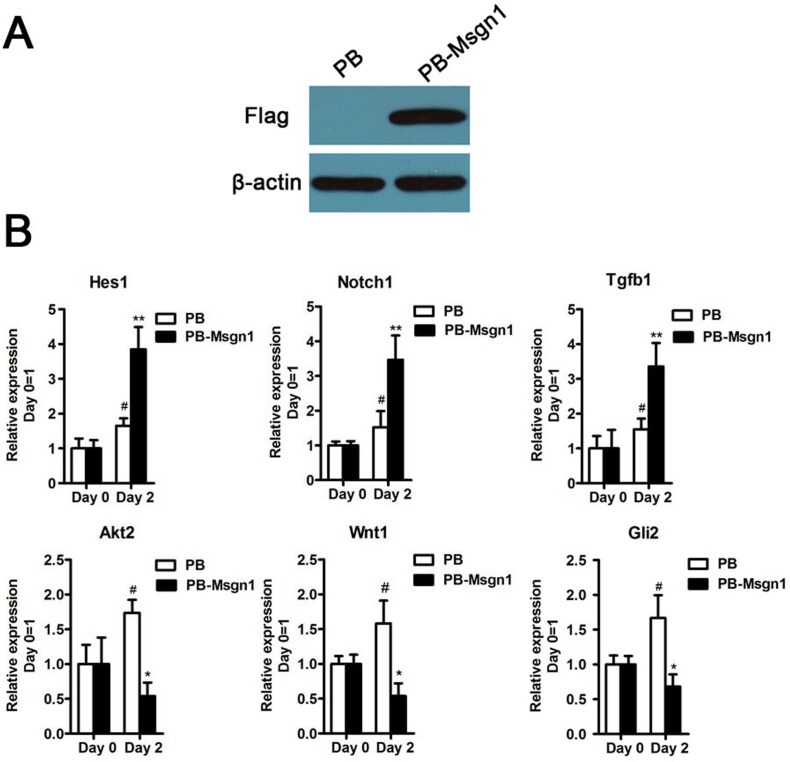
The validation results of qRT-PCR (**A**) Flag-tagged Msgn1 was introduced into 46C mESCs and the protein level of Flag-tagged Msgn1 was determined by Western blot. β-actin was used as a loading control. (**B**) qRT-PCR analysis of the indicated gene expression for the indicated time in mESCs and mESCs-derived EBs. Data represent mean±s.d. of three biological replicates. #p < 0.05 vs PB:Day 0. *p < 0.05, **p<0.01 vs PB-Mgsn1:Day 0.

To further examine whether *Msgn1* could regulate these genes expressions during mESC differentiation, we generated a flag-tagged *Msgn1*-overexpressing mESC line using a PiggyBac vector (PB-Msgn1) in which *Msgn1* expression was efficiently enhanced (Figure 5A). According to our quantitative real-time PCR (qRT-PCR)results, the expression levels of above six genes at day 2 EBs could be detected. As shown in Figure 5B, the overexpression of *Msgn1* was able to induce higher expression levels of *Hes1*, *Notch1* and *Tgfβ1* than the PB empty vector, while inhibiting the expressions of *Wnt1*, *Akt2* and *Gli2*. The validation of our qRT-PCR results was consistent with the previous gene expression profiles.

## MATERIALS AND METHODS

### Microarray data collection and preprocessing

First of all, we searched GEO (www.ncbi.nlm.nih.gov/geo/) for the gene expression profiling studies related to Msgn1 overexpression. Data sets were included in our reanalysis if they met the following conditions: (1) the data were genome-wide, (2) Msgn1 over expression datasets and Msgn1 normal expression datasets were compared, (3) complete microarray raw or normalized data were available. Then, we got the dataset of GSE29848, contributed by Ravindra et al. [34]. In this data set, inducible A2lox-Flag Msgn1 ES cells were differentiated to form embryoid bodies (EBs) for 2 days. Flag-Msgn1 was induced on day 2 with doxycycline(DOX) and samples were collected at three time points, 12h, 24h and 48h after the addition of doxycycline (Table 2). Uninduced cells were used as controls, and experiments were performed in triplicate.

**Table 2:** The summary of gene expression datasets

Series	Contributor	Platform	Design		Replicates
			Time Points	Treatment	
GSE29848	Chalamalasetty et al.	Affymetrix Mouse Genome 430 2.0 Array	12h	MinDox	3
				PlusDox	3
			24h	MinDox	3
				PlusDox	3
			48h	MinDox	3
				PlusDox	3

It showed the summary of experiment design of dataset of GSE29848 used in our study. In Chalamalasetty et al.'s study, Msgn1 was induced with doxycycline (PlusDox) and uninduced cells (MinDox) were used as controls. Samples were collected at three different time points including 12h, 24h and 48h. Experiments were performed in triplicate.

In order to assess the effect of preprocessing on the comparison, data preprocessing was performed using software packages developed in Bioconductor ver. 2.6.0 and R ver. 2.10.1. Each Affymetrix data set was background-adjusted. Normalized log2 probe-set intensities were calculated using the Robust Multichip Averaging (RMA) algorithm in the Affy package [35].

### Gene set enrichment analysis

Our GSEA of pathways and genes was performed using the Category package in Bioconductor ver. 2.6.0 [36]. The target of GSEA is to reach a verdict as to whether the members of a gene set S are randomly distributed throughout the whole reference gene list L or are found primarily at the top or bottom of L. The relative robustness to noise and outliers in the data are remarkable advantages of GSEA. In our analysis, the gene sets of fewer than 10 genes were excluded. The t-statistic mean of the genes was calculated in each KEGG (Kyoto Encyclopedia of Genes and Genomes) pathway. The threshold of the significance level p-values was chosen as 0.01 for the most significant pathways correlated with WD using a permutation test 1,000 times. Accordingly, the significant pathways and genes were then identified when comparing Msgn1overexpression and wildtype mice. The following category of identified signaling pathways is rooted in the KEGG pathway map br08901 of BRITE Functional Hierarchies in the KEGG database (http://www.genome.jp/keggbin/get_htext?br08901.keg). The annotation of significant genes in each signaling pathway was implemented by using the biomaRt package (http://www.biomart.org/) BioMart ver. 0.8 rc3 (version 0.8 of release candidate 3).

### Regulatory elements and transcription factors of coregulated genes

To predict common regulatory elements (REs) for our input genes, we used a web server called DiRE (Distant Regulatory Elements of coexpressed genes, http://dire.dcode.org/), which is based on the Enhancer Identification (EI) method for predicting distant regulatory elements in higher eukaryotic genomes. DiRE predicts function-specific REs and scores the association of individual transcription factors (TFs) with the biological function shared by the group of input genes [37]. We selected a random set of 5,000 genes in the genome of mm9 (Mus musculus9) as the source of background genes. TF occurrence for the centage of candidate REs which contain a conserved binding site for a special TF and TF importance for the product of TF occurrence and TF weight were two major parameters of our predicted TFs. The candidate associated TFs with the value of TF importance more than 0.05 would be selected.

### Cell culturing and differentiation

46C mouse embryonic stem cells (mESCs) were provided by Qi-Long Ying (University of Southern California), which were cultured on 0.1% gelatin-coated dishes. mESC medium contains DMEM(Sigma), 10% FBS (HyClone), 1× MEM nonessential amino acids (Invitrogen), 1× L-glutamine (Invitrogen),1× penicillin and streptomycin (Sigma), 0.01 mM β-mercaptoethanol (Invitrogen) and 1000 units/ml LIF(Millipore). mESCs were differentiated by forming embryoid bodies(EB) in the mESC medium without LIF.

### Plasmid construction and cell transfection

The coding sequence of Mesogenin1(*Msgn1*) was inserted into a PiggyBac (PB) vector. The recombinant plasmid was then transduced into mESCs combined with 2 μg transposase using LTX (Invitrogen). After transfection, mESCs were selected with 2 μg/ml puromycin for 24 hours to obtain positive clones.

### Western blotting

mESCs were lysed with RIPA buffer. Bradford (Bio-Rad) assay was used to determine the protein concentration. The proteins were separated with a 10% PAGE gel and electrotransferred onto a PVDF membrane. Probing was performed with specific primary antibodies and HRP-conjugated secondary antibodies. The primary antibodies used were FLAG (Sigma) and β-actin (Santa Cruz Biotechnology).

### Quantitative real-time PCR (qRT-PCR)

Total RNA was extracted from the cells using TRIzol. cDNA was synthesized from 2 μg total RNA using reverse transcriptase (Takara) according to the manufacturer's instructions. Primers were designed using Primer 3.0 input software and are listed in Table 3. qRT-PCR was performed with TransStart Tip Green qPCR SuperMix (Takara). The expression level of each transcript was normalized with glyceraldehyde-3- phosphate dehydrogenase (GAPDH) and analyzed using the 2^−ΔΔCt^ method.

**Table 3:** The sequences of target genes primers for RT-PCR

Genes	Forward(5′→3′)	Reverse(5′→3′)
Hes1	gagtgcatgaacgaggtgac	cgttgatctgggtcatgcag
Notch1	aggctacacaggaagcatgt	cgatgccatcctcacaagtg
Tgfβ1	cgcaacaacgccatctatga	actgcttcccgaatgtctga
Wnt1	gctgcagtgacaacatcgat	cttggcgcatctcagagaac
Akt2	acagccctcaagtatgcctt	accacatctctcgagtgcaa
Gli2	atgccaaccagaacaagcag	tcttgaccttgctccgctta

## SUPPLEMENTARY MATERIAL












