# Supplementary material for: A comprehensive transcriptomic analysis of differentiating embryonic stem cells in response to the overexpression of Mesogenin 1
Source: Aging (Albany NY). 2016 Oct 6;8(10):2324–35. doi: 10.18632/aging.101049 (PMC5115891; doi:10.18632/aging.101049)
Supplement: Supplementary file 1 [file aging-08-2324-s001.pdf]

SUPPLEMENTARY MATERIAL

Please browse the links in Full Text verions to see the Supplementary Tables:

Table S-1. The significantly regulated pathways during somitogenesis under Msgn1 overexpression. The details of all identified pathways according to GSEA of the data sets of 18 samples used in the microarray experiment by the comparison of Msgn1 overexpression to wild-type mice over a 48 hour timecourse were showed, which respectively includes pathways, functions, the number of genes , pvals, the number of TFs. There were 100 significant pathways identified with $p < 0.01$ at 12h, including 83 upregulated and 17 downregulated pathways. Similarly, 113 significant pathways, including 29 upregulated and 84downregulated ones at 24h, and 183 significant pathways, including 50 upregulated and 113 downregulated ones at 48h, were identified.

Table S-2. The significantly regulated TFs during somitogenesis under Msgn1 overexpression. TFs potentially involved in embryonic stem cells differentiating in the case of Msgn1overexpression over a 48 hour timecourse. The table showed associated TFs with potential target genes which are co-regulated in each of above-mentioned 376 pathways and cutoff value of TF importance. Abbreviations: TFs, transcription factors.

Table S-3. The overlapping genes between microarray and ChIP-seq results in 12h, 24h and 48h.

Table S-4 The distribution of signaling pathways at three points. Signaling pathways during somitogenesis under Msgn1 overexpression and KEGG pathway maps of signaling pathways at three points were showed. There were 1 signaling pathway only significantly regulated in 12h, 2 signaling pathways only significantly regulated in 24h and 2 signaling pathways only significantly regulated in 48h. 2 signaling pathways played roles at both 12h and 24h. 5 signaling pathways regulated significantly at 24h and 48h, 3 signaling pathways regulated significantly at 12h and 48h, 9 signaling pathways regulated at three different time points.

Table S-5. The associated genes in each significant pathways during somitogenesis under Msgn1 overexpression. The associated genes in each significant pathway during somitogenesis under Msgn1 overexpression were showed. Data was performed using software packages developed in Bioconductor ver. 2.6.0 and R ver. 2.10.1. ID and gene symbol of 83 upregulated and 17 downregulated pathways at 24h, 29 upregulated and 84downregulated ones at 24h, 50 upregulated and 113 downregulated ones at 48h were listed.
